# Microsomal epoxide hydrolase gene polymorphisms and risk of chronic obstructive pulmonary disease: A comprehensive meta-analysis

**DOI:** 10.3892/ol.2012.1099

**Published:** 2012-12-28

**Authors:** HUI LI, WEI-PING FU, ZE-HUI HONG

**Affiliations:** 1 Department of Genetics and Developmental Biology, Southeast University School of Medicine; Nanjing 210009;; 2 The Key Laboratory of Developmental Genes and Human Disease, Ministry of Education, Southeast University, Nanjing 210009;; 3 Department of Respiratory Critical Care Medicine, The First Affiliated Hospital of Kunming Medical College, Kunming 650091, P.R. China

**Keywords:** microsomal epoxide hydrolase gene, chronic obstructive pulmonary disease, polymorphism, meta-analysis

## Abstract

Microsomal epoxide hydrolase (EPHX1) is an enzyme involved in the detoxification the products of smoking and is proposed to be a genetic factor for the development of chronic obstructive pulmonary disease (COPD). Two functional polymorphisms of EPHX1, T113C and A139G, have been analyzed in numerous studies to assess the COPD risk attributed to these variants. However, the conclusions were controversial. We performed a comprehensive meta-analysis to clarify these findings. A total of 24 studies comprising 8,259 COPD patients and 42,883 controls were included. The overall results showed that the EPHX1 113 mutant homozygote was significantly associated with an increased risk of COPD (OR, 1.33; 95% CI, 1.06–1.69). The subgroup analyses demonstrated this association in Caucasian individuals (OR, 1.61; 95% CI, 1.12–2.31) but not in Asian individuals. The 139 mutant heterozygote was significantly associated with a decreased risk of COPD in Asian populations (OR, 0.82; 95% CI, 0.68–0.99) but not in Caucasian populations. Pooled analyses revealed that the extremely slow (OR, 1.77; 95% CI, 1.23–2.55) and slow EPHX1 enzyme activity (OR, 1.44; 95% CI, 1.13–1.85) were associated with an increased risk of COPD, while the fast enzyme activity was not associated with a decreased risk of COPD. The stratified analysis demonstrated this association in Caucasian but not in Asian individuals. Furthermore, a modest difference in the risk of COPD was observed between the subgroups by using the cigarette smokers or the non-smokers as controls. A significant correlation between the two functional polymorphisms, T113C and A139G, of the EPHX1 gene and the enzyme activity and the individual’s susceptibility to COPD was noted. In addition, the results supported a contribution of EPHX1 to the aetiology of COPD.

## Introduction

Chronic obstructive pulmonary disease (COPD) is a complex disease characterized by irreversible airflow limitation, abnormal permanent distal air-space enlargement and emphysema in the lung. This is usually progressive and associated with an abnormal inflammatory response of the lungs to noxious particles or gases (1). COPD is a major and increasing cause of morbidity and mortality worldwide (2). The World Health Organization has predicted that it is likely to be the third leading cause of mortality in the world by the year 2020. Although cigarette smoking is widely accepted as the most significant risk factor for COPD, only 10–15% of smokers develop the disease (3). This, together with the familial clustering of early-onset COPD (4), as well as a susceptibility to frequent exacerbation in these individuals (5), strongly suggests a genetic factor in the pathogenesis of COPD.

As the only well-defined genetic factor of COPD, α1-antitrypsin deficiency, is rare in worldwide populations, other genetic factors must be involved in the susceptibility and development of COPD. Given that 95% of those who develop COPD are smokers, oxidative stress caused by smoking is considered to be a pathogenetic factor for COPD. Thus, the genes encoding enzymes that protect the lung against smoke-induced oxidative stress have received more attention and become the focus of genetic COPD studies. Microsomal epoxide hydrolase (EPHX1) is an enzyme essential for the metabolism of the highly reactive epoxide intermediates produced by cigarette smoke and is expressed at varying levels in the majority of tissues and cell types (6). Epidemiological studies have shown that EPHX1 activity in the liver, lung and peripheral blood leucocytes varies as much as 50-fold in Caucasian populations (7). The human EPHX1 gene is located on chromosome 1q42.1 and consists of nine exons. Further studies have reported that two common polymorphisms in the coding regions are responsible for the variable enzyme activity, conferring decreased activity by one mutation and increased activity by another (8). The T to C transition in exon 3 changes tyrosine (Tyr) residue 113 to histidine (His), thus reducing the enzyme activity by ∼50% (slow allele). The second, the A to G transition in exon 4 changes histidine (His) residue 139 to arginine (Arg) and produces an enzyme with an activity increased by ∼25% (fast allele) (8). Based on these two single nucleotide polymorphisms (SNPs), the population may be divided into four groups of putative EPHX1 phenotypes with various activities (fast, normal, slow and extremely slow) (8,9).

Since the initial report of an association between increased susceptibility to COPD and EPHX1 113 His-His homozygosity in Caucasian populations (9), a number of studies have been performed to assess the association between the EPHX1 polymorphisms and COPD in various populations (10–32). However, the results of the studies remain controversial and inconclusive. A number of studies supported this association (12,13,18,19,22,24,26,27,29) or reported an association with disease severity rather than susceptibility (14,30), while others did not observe this association (10,11,20,21,23,24,31). The remaining studies reported a protective effect for this polymorphism in the development of COPD (16,17). The lack of replication and consistency in these studies occured, most likely, due to the different ethnic populations used in the various studies, the poor matching of cases and controls or the small sample sizes lacking the statistical power to produce a reliable conclusion in any individual study. Thus, a comprehensive analysis is essential. A systematic review and meta-analysis of the published data may be a powerful tool to aid the elucidation of this association. Although three meta-analyses have been performed to attempt to clarify this question, two completely different and contradictory conclusions have been reported. Two studies reported that the EPHX1 113 mutant homozygote was a risk factor for COPD (26,33), while the other reported it to have a protective effect (17). Since then, additional studies of the association between EPHX1 polymorphisms and COPD have been reported.

In the present study, a new comprehensive meta-analysis was performed to systematically investigate the association between the EPHX1 polymorphisms and an individual’s susceptibility to COPD. A total of 24 published case-control studies were included in this meta-analysis. The putative EPHX1 enzyme activity and risk of COPD were predicted by single polymorphism of T113C/A139G and combined double polymorphisms. Analyses stratified by ethnicity, sample size, cigarette smoking status of the controls and Hardy-Weinberg equilibrium (HWE) violation of the controls were performed to explore the variations which affect the final conclusion.

## Materials and methods

### 

#### Literature search

A literature search was performed in PubMed, MEDLINE, Embase, Wanfang Database and Weipu Database for studies published up to April 2012 to use in the present meta-analysis. The key terms were ‘EPHX1 or microsomal epoxide hydrolase or EPHX or EPOX or HYL1 or MEH’, ‘COPD or chronic obstructive pulmonary disease’ and ‘polymorphism’ in various combinations. The search was limited to articles published in English and Chinese. References matching the above criteria were also searched manually to identify additional studies.

#### Inclusion and exclusion criteria

The eligible investigations met the following criteria: i) the studies were case-control studies designed to explore the association between at least one of the two EPHX1 gene polymorphisms (T113C and A139G) and COPD susceptibility; ii) the studies provided data on the distribution of EPHX1 gene polymorphisms in the case-control population; iii) the studies were published in the English or Chinese language. Studies were excluded if they did not contain enough data for meta-analysis, were abstracts or reviews or were duplicated within other included studies.

#### Data extraction

Two authors searched the studies and extracted the data according to the above inclusion and exclusion criteria. The following characteristics were collected from the eligible studies: first author, year of publication, country of studied population, ethnicity, sample size of the cohorts, number of COPD patients, number of controls, source of the control group (population-based or hospital-based) and the EPHX1 gene T113C and A139G allele and genotype distributions among the case and control groups. Different case-control groups within one study were considered to be independent studies. The cigarette smoking status of the controls was strategically classified as current smokers, ex-smokers and non-smokers.

#### Statistical analyses

ORs with 95% CIs were calculated to assess the association between EPHX1 gene polymorphisms and COPD risk. The wild type homozygotes, TT of T113C and AA of A139G, were used as reference genotypes. Thus the comparisons were mutant homozygote or heterozygote vs. reference genotype (CC vs. TT, CT vs. TT, GG vs. AA and AG vs. AA ). The ORs of COPD risk associated with EPHX1 enzymatic activity were estimated using the normal activity as the reference group (extemely slow vs. normal, slow vs. normal and fast vs. normal). Stratified analyses were performed using ethnicity, sample size, cigarette smoking status of the controls and HWE violation of the controls.

Heterogeneity across the publications was assessed with the Cochran’s χ^2^ test (Q-test) (34) and P<0.10 was considered to indicate statistically significant heterogeneity. The I^2^ test was also performed to evaluate heterogeneity between studies. A high heterogeneity was considered to be present when I^2^>50% and much higher when I^2^>75% (35). A higher heterogeneity is a common phenomenon in genetic association studies (36). The pooled OR was calculated by a fixed-effect model (using the Mantel-Haenszel method) or a random-effect model (using the DerSimonian-Laird method) according to the heterogeneity among the studies (37,38). The statistical significance of the ORs was analyzed by the Z test and P<0.05 was considered to indicate statistically significant differences. Random effect meta-regression models with restricted maximum likelihood estimation were performed to evaluate the variance among the individual ORs when heterogeneity was detected. The pre-specified possible sources of inter-study heterogeneity were: ethnicity of a population, source of the control group, sample size and HWE violation. Publication bias was assessed using a funnel plot and Egger’s and Begg’s tests (39,40). The funnel plot was asymmetrical when there was evidence of publication bias.

Statistical analyses were performed using the Revman 5.1 software and STATA 10.0 software. The P-value was two-tailed and P<0.05 was considered to indicate a statistically significant difference.

## Results

### 

#### Study characteristics

**Figure 1:**
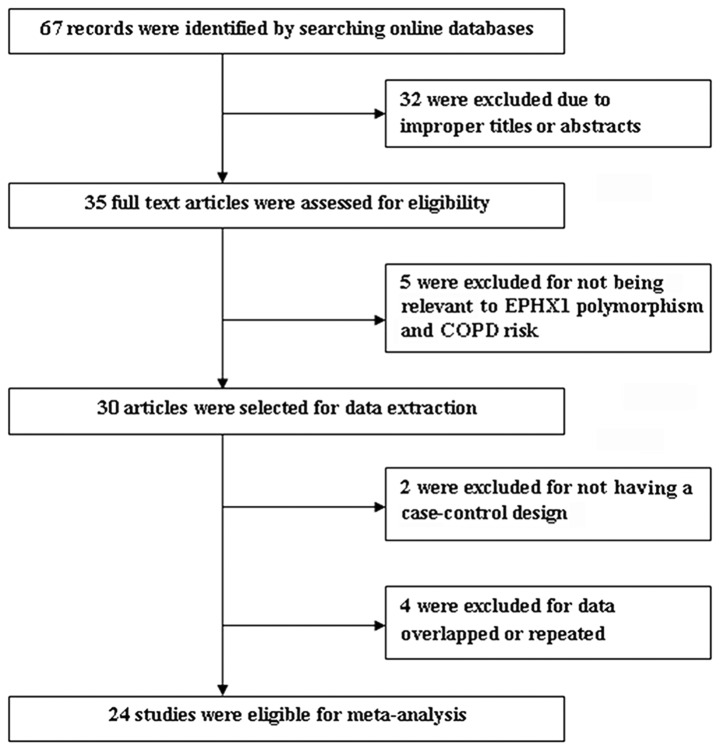
Flow diagram of the study selection for the meta-analysis.

As shown in Fig. 1, a total of 67 results were identified after an initial search of the selected electronic databases. After screening the titles and abstracts, 35 articles were selected for further review. Among them, five studies were excluded for not referring to the association between the EPHX1 gene polymorphism and COPD risk; two were excluded since they were not case-control studies between the EPHX1 gene polymorphism and COPD risk; and four were excluded due to the data overlapping with that of another study. Thus, a total of 24 studies were suitable for meta-analysis (9–32). Among them, 11 studies were performed in Caucasian populations (9,12,13,16,17,22,23,25,26,29,31), 12 studies were performed in Asian populations (10,11,14,15,18–21,27,28,30,32) and one was performed in an African population (24). The article by Smith and Harrison had two independent studies and thus these were considered separately (9). The study by Chen *et al* evaluated only the association between the EPHX1 113 T/C gene polymorphism and COPD risk (27). The total number of samples involved in the 24 eligible studies was 51,142, which included 8,259 COPD patients and 42,883 controls. The HWE test had been calculated for the T113C and A139G polymorphisms in the control groups for all the included 24 papers. For the T113C locus, 15 studies (10,14,16,17,20,22–31) obeyed the HWE and 10 (9,11–13,15,18,19,21,32) deviated. For the A139G locus, with the exception of three studies (18,21,28), the remaining 21 studies (9–17,19,20,22–26,29–32,36) obeyed the HWE. Among the overall studies, 17 (9,10,12,13,15,18–20,22–26,28,29,31) further evaluated the putative EPHX1 enzyme activity and COPD risk using the method described by Hassett *et al*(8). The characteristics of each study are presented in Table I.

### Quantitative data synthesis

#### EPHX1 113 mutant homozygote and COPD risk

**Figure 2:**
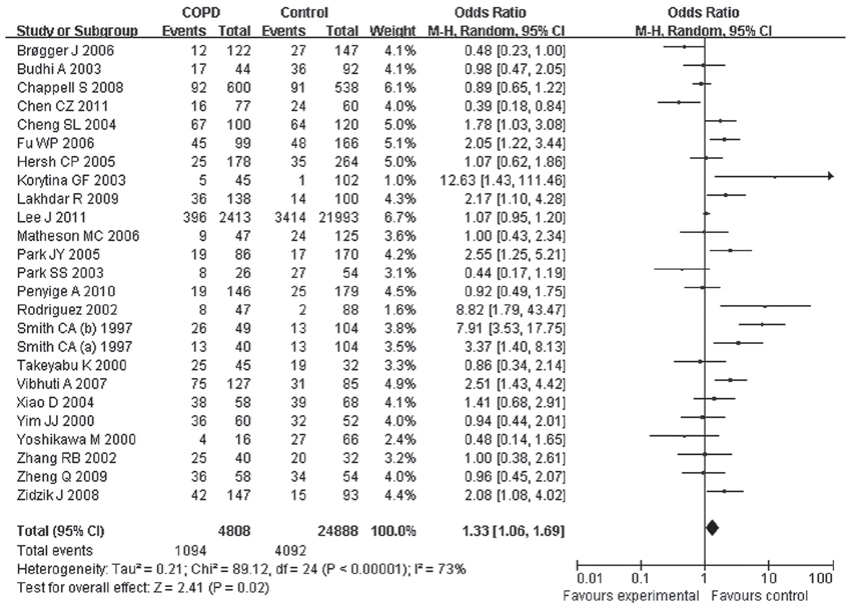
Forest plot describing the association of the EPHX1 113 mutant homozygote and COPD risk. EPHX1, microsomal epoxide hydrolase; COPD, chronic obstructive pulmonary disease.

After pooling the data from the 25 studies (with Smith and Harrison’s considered as two separate studies) for meta-analysis, the associations between the EPHX1 113 mutant homozygote and heterozygote and the COPD risk were analyzed. Summary ORs are shown in Table II. The overall OR showed that the 113 mutant homozygote was significantly associated with an increased risk of COPD (OR, 1.33; 95% CI, 1.06–1.69). Heterogeneity existed among the studies (P<0.0001; I^2^, 73%), so a random-effect model was used for the analysis (Fig. 2).

In the analysis stratified by ethnicity, the study populations were divided into two subgroups, one comprising Asian individuals (12 studies) and the comprising Caucasian individuals (12 studies). The pooled ORs showed a significant association between the 113 mutant homozygote and COPD risk in the Caucasian subgroup (OR, 1.61; 95% CI, 1.12–2.31), but this was not significant in Asian subgroup (OR, 1.07; 95% CI, 0.76–1.52). For sample size, the studies were stratified into two subgroups, one comprising studies with >200 subjects and one with <200 subjects. In these subgroups, a significant association between the 113 mutant homozygote and COPD risk was observed in the >200 subject subgroup (OR, 1.54, 95% CI, 1.17–2.02), but not in the <200 subgroup (OR, 0.80, 95% CI, 0.55–1.15; Table II).

For case-control studies, a deviation from the HWE of the control groups may lead to an erroneous result and imply insufficient random sampling. Thus the 15 studies in which the controls were in HWE were analyzed. Six studies were in Asian populations, eight populations were Caucasian and one was African. There were significant heterogeneities among the populations (P=0.004; I^2^, 56%). The pooled OR by the random-effects model showed no significant association between the 113 mutant homozygote and COPD risk (OR, 1.07; 95% CI, 0.86–1.34). When these 15 studies were stratified by ethnicity, no statistically significant associations were detected (Asian: OR, 0.86; 95% CI, 0.52–1.43; Caucasian: OR, 1.09; 95% CI, 0.85–1.39).

Analysis stratified by the cigarette smoking status of the controls was performed. The controls were strategically classified as current smokers, ex-smokers and non-smokers. A moderate difference was observed between the cigarette smokers and non-smokers (Table II). The OR for the 113 mutant homozygote vs. wildtype homozygote in non-smokers was 1.86 (95% CI, 1.14–3.04) and 1.11 (95% CI, 0.84–1.48) in smokers.

#### EPHX1 113 mutant heterozygote and COPD risk

In the genetic model of the 113 mutant heterozygote vs. wildtype homozygote, 25 studies were pooled by the random-effects model since heterogenetity existed among the studies (P<0.0001; I^2^, 69%). Overall, the OR was 1.12 (95% CI, 0.96–1.30) and was not significant (Table II), which suggested that the 113 mutant heterozygote was not associated with COPD risk. Analyses stratified by ethnicity, sample size, smoking status of the controls and HWE violation of the controls were performed to further examine the association between the 113 mutant heterozygote and COPD risk. The results are presented in Table III. All the results of the stratified analyses were consistent with the main analysis.

#### EPHX1 139 mutant homozygote and COPD risk

A total of 24 studies detected an association between the 139 mutant homozygote and COPD risk. However, meta-analysis of these studies did not suggest an association between the 139 mutant homozygote and COPD risk (OR, 0.90; 95% CI, 0.79–1.02) and there was no heterogeneity among the studies (P= 0.67; I^2^, 0%). Stratifying by ethnicity, sample size, smoking status of the controls and HWE violation of the controls also showed no association.

#### EPHX1 139 mutant heterozygote and COPD risk

A total of 24 studies detected the association between the 139 mutant heterozygote and COPD risk. The overall OR showed that the 139 mutant heterozygote was not significantly associated with COPD risk (OR, 0.96; 95% CI, 0.91–1.01) and there was a low heterogeneity among the studies (P= 0.15; I^2^, 3%). Stratification by ethnicity demonstrated that there was no significant association in Caucasian populations (OR, 0.99; 95% CI, 0.88–1.11) but revealed a statistically marginally significant association in Asian populations (OR, 0.82; 95% CI, 0.68–0.99).

#### Putative EPHX1 enzyme activity and COPD risk

**Figure 3:**
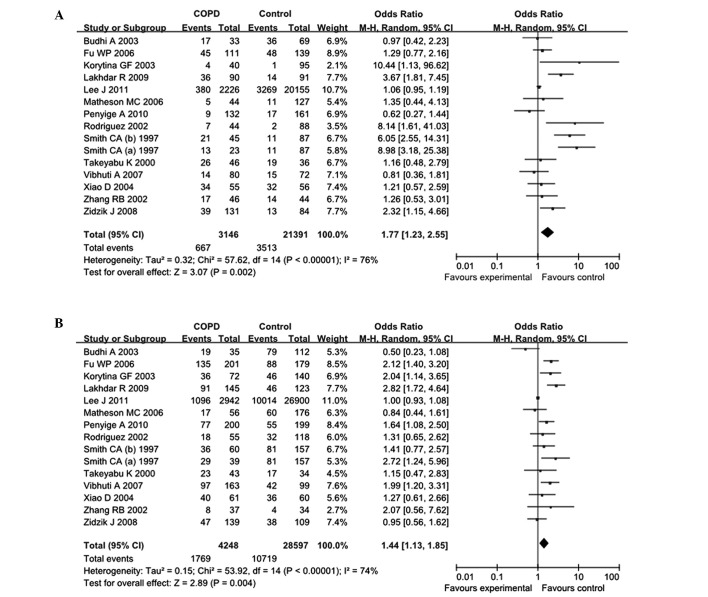
Forest plot describing the association of putative EPHX1 enzyme activities and COPD risk. (A) Putative extemely slow vs. normal enzyme activity, and (B) putative slow vs. normal enzyme activity predicted by genotype of polymorphisms T113C/A139G and COPD risk. EPHX1, microsomal epoxide hydrolase; COPD, chronic obstructive pulmonary disease.

In order to evaluate the association of these two functional polymorphisms and their enzyme activity with COPD risk, the association of EPHX1 enzyme activity predicted by the genotype combination of T113C and A139G polymorphisms with COPD risk was analyzed. In the overall comparisons with the putative normal EPHX1 enzyme activity, the extremely slow EPHX1 enzyme activity (OR, 1.77; 95% CI, 1.23–2.55) and slow EPHX1 enzyme activity (OR, 1.44; 95% CI, 1.13–1.85) increased the COPD risk significantly, while the fast EPHX1 enzyme activity did not affect the COPD risk (OR, 1.03; 95% CI, 0.87–1.21; Table III and Fig. 3).

In the analyses stratified by ethnicity, the increased COPD risk of extremely slow and slow EPHX1 enzyme activity was observed in the Caucasian populations (extremely slow enzyme activity: OR, 2.64; 95% CI, 1.30–5.38; slow enzyme activity: OR, 1.31; 95% CI, 1.01–1.71), but not in the Asian populations (extremely slow enzyme activity: OR, 0.1.14; 95% CI, 0.84–1.54; slow enzyme activity: OR, 1.41; 95% CI, 0.90–2.19). In the further assessments of the analyses stratified by sample size, smoking status of the controls and HWE violation of the controls, the extremely slow enzyme activity showed an increased COPD risk in the >200 subjects subgroup (OR, 1.90; 95% CI, 1.25–2.88) and the subgroup with the non–smokers as controls (OR, 2.95; 95% CI, 1.56–5.56). For the stratified analyses using the genetic model of slow enzyme activity vs. normal enzyme activity, the increased COPD risk was also observed in the >200 subjects subgroup (OR, 1.44; 95% CI, 1.11–1.88). Notably, the increased COPD risk of the slow enzyme activity was observed not only in the subgroup with the non-smokers as controls (OR, 1.42; 955CI=1.04–1.93) but also in the subgroup with the smokers and ex-smokers as controls (OR, 1.49; 95% CI, 1.01–2,21; Table III).

#### Publication bias

**Figure 4:**
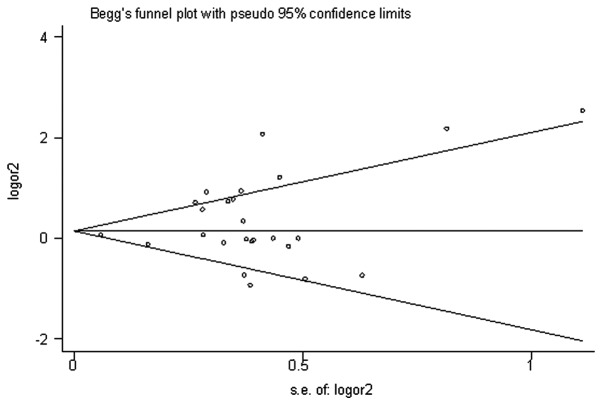
Begg’s funnel plot for the publication bias in the selection of studies on the EPHX1 T113C polymorphism (TT vs. CC). EPHX1, microsomal epoxide hydrolase.

The Begg’s funnel plots and Egger’s tests were performed to assess the potential publication bias. The shape of the Begg’s funnel plots appeared to be symmetrical in the 113 mutant homozygote vs. wildtype homozygote (113 CC vs. TT) genetic model (Fig. 4). Neither the Begg’s test (P= 0.907) nor Egger’s test (P= 0.158) indicated any statistically significantly evidence of publication bias. Moreover, evaluations of publication bias for the other genetic models did not reveal any significant results (data not shown).

## Discussion

The EPHX1 gene encodes a xenobiotic metabolizing enzyme that detoxifies the reactive epoxides produced by smoking to form water and soluble dihydrodiol compounds and is widely expressed in the bronchial epithelium. Thus it has been a significant focus for studies concerning genetic susceptibility to COPD over the past decade. The genetic polymorphisms T113C and A139G in the coding regions have been observed to alter the enzyme activity and thus, are considered to affect the individual’s susceptibility to COPD or risk of developing COPD. However, studies of genetic polymorphisms of EPHX1 in COPD have provided different and inconclusive results.

The explanations for this may be the different ethnic populations used in the various studies, relatively small sample sizes lacking the statistical power to produce a reliable conclusion for any individual study or mismatching for age, gender and smoking history in the controls. A perfect COPD genetic association study would be large and longitudinal, using the cumulative reduction in lung function in relation to cumulative smoking as a main outcome measure. Such a study would take decades to complete and require tens of thousands of subjects. Thus, to avoid these error sources, meta-analysis studies appear to be an essential tool for summarizing case-control studies.

The present meta-analysis provides the most comprehensive and up-to-date evidence on the EPHX1 genetic polymorphisms T113C and A139G, enzyme activity resulting from these two genetic polymorphisms and the risk of developing COPD. A total of 25 case-control association studies with 8,259 COPD patients and 42,883 controls were included in the present meta-analysis. Through systematic analyses, the 113 mutant homozygote was demonstrated to be significantly associated with an increased risk of COPD. This result contrasted with the study by Brøgger *et al*, in which a reduced risk of COPD was detected in the 113 mutant homozygotes (17). The analyses stratified by ethnicity showed the significant increase in risk existed only in the Caucasian populations and not in the Asian populations. These results were the opposite of those of the study by Hu *et al*, in which an increased risk of COPD was observed in Asian populations, not in Caucasian populations (33). The results are also inconsistent with the study by Lee *et al*, in which the 113 mutant homozygote was not associated with CODP either in Asian or Caucasian populations (26). The reasons for this discrepancy may be the different studied populations and inclusion criteria of COPD phenotypes in the previous meta-analyses. As shown in Table II, the association was only demonstrated in analyses stratified by combining studies with a sample size >200 subjects, which emphasized the importance of having sufficient power with a large enough sample size. In the analyses stratified by the cigarette smoking status of the controls, a modest difference in the risk of COPD was detected between cigarette smokers and non-smokers. Notably, the increased risk of COPD was only detected in the studies with non-smokers as controls. As is known, smoking is the main risk factor for COPD and associated types of cancer, such as lung cancer. However, these diseases are the outcome of genes and environmental interactions and the pathogeneses are complex and remain unclear. In a previous study, these T113C and A139G polymorphisms of the EPHX1 gene varied from being risk factors to protective factors according to the cigarette smoking status of the lung cancer patients, whereby the low activity genotype of the EPHX1 gene was a risk factor for non-smokers, but a protective factors for heavy smokers (41). In the study by Xiao *et al*, the authors also noted that when the COPD patients were non-smokers, the low activity genotype was a risk factor, but became a protective factor when the COPD patients were smokers (15). Thus in the present study, whether the observed association was real or spurious could not be decided due to the lack of detailed data, such as the number of packs smoked per year and smoking history of the COPD patients and controls in the included studies. To confirm this susceptibility association, more studies containing the above information for patients and controls are required in the future. The 113 mutant heterozygote was not associated with an increased COPD risk. The 139 mutant heterozygote was significantly associated with a decreased risk of COPD in Asian populations, but not in the Caucasian population.

When evaluating the EPHX1 enzyme activity and the risk of COPD, the extremely slow activity phenotype and slow activity phenotype of EPHX1 were revealed to be significantly associated with an increased risk of COPD. The stratified analyses demonstrated the association in Caucasian populations, studies with >200 subjects and studies with non-smokers as the controls. These findings are consistent with the known role of the EPHX1 enzyme in the detoxification of harmful epoxides from smoking and its involvement in the first-pass metabolism of smoking-induced highly reactive epoxide intermediates (42). Regardless of the 113 mutant homozygote, extremely slow activity or slow activity, they all exhibited decreased EPHX1 enzyme activity and thus increased the risk of developing COPD in populations. In summary, these results suggest that the two polymorphisms, T113C and A139G, of EPHX1 were associated with the risk of COPD not only in statistical calculations, but also at physiological function levels.

Although meta-analysis is a powerful statistical method, the inherent limitations of the present study should be addressed. Thus, in interpreting the results, caution should be taken. First, relatively large heterogeneity existed in the 113 mutant homozygote vs. wildtype homozygote meta-analysis. Analyses stratified by ethnicity, sample size, cigarette smoking status of the controls and HWE violation of the controls did not significantly reduce the heterogeneity. The heterogeneity may be caused by the different criteria in the selection of COPD patients and controls, including age and gender distributions and lifestyle factors. However, the lack of original data for the included studies limited any further evaluation. Second, only studies written in English and Chinese were included and related studies in other languages were not included, which may bias the conclusion of the present study. Third, publication bias may not be excluded, although the tests showed negative results. Studies reporting significant associations would be more readily published, while studies with non-significant associations would be more difficult to publish. Fourth, although half of the included studies were performed in Asian populations, the total size of the Asian populations was relatively small compared with the Caucasian populations. In the analyses stratified by ethnicity, the detected associations were only demonstrated in the Caucasian populations, not in the Asian populations. As the scale of the effect of EPHX1 variants on the risk of COPD may depend on ethnicity (43), more studies performed in Asian populations are required to test the present conclusions. Fifth, gene-gene and gene-environment interactions may affect an individual’s susceptibility to COPD. In a complex polygenic disease such as COPD, it is likely that multiple genes are operating and the genetic susceptibility may be dependent on the coincidence of several genetic polymorphisms acting together. The polymorphism of each gene may therefore confer only a small relative risk of COPD and it is likely that the coincidence of numerous polymorphisms are important in its pathogenesis, although there was not enough data to eliminate these interfering factors. In addition, although smoking is a significant risk factor for developing COPD, only a limited amount of detailed information such as packs per year and smoking history, was available in the studies so the corresponding stratified analyses were not performed. These may bias the present conclusions. Well-designed studies containing this information are required. Sixth, as the majority of studies did not mention potential population stratification of patients and controls, it is not possible to rule out a role for population structure in the observed association.

In conclusion, the present meta-analysis is the most comprehensive and up-to-date appraisal of the two EPHX1 genetic polymorphisms, T113C and A139G, enzyme activity and the risk of developing COPD. The results indicated that they were genetic factors for the susceptibility of an individual to COPD. Further well-designed studies of various ethnic populations should be performed to evaluate these associations.

## Figures and Tables

**Table I:** Characteristics of the studies included in the meta-analysis.

First author (ref)	Year	Country	Ethnicity	Case	Control	Genotyping Method
Smith CA (9)	1997	UK	Caucasian	68	203	PCR-RFLP
Smith CA (9)	1997	UK	Caucasian	94	203	PCR-RFLP
Takeyabu K (10)	2000	Japan	Asian	79	58	Sequencing
Yim JJ (11)	2000	Korea	Asian	83	76	PCR-RFLP
Yoshikawa M (30)	2000	Japan	Asian	40	140	PCR-RFLP
Rodriguez F (12)	2002	Spain	Caucasian	79	146	PCR-RFLP and SSCP
Zhang RB (20)	2002	China	Asian	55	52	PCR-RFLP
Budhi A (28)	2003	Japan	Asian	63	172	PCR-RFLP
Korytina GF (13)	2003	Russia	Caucasian	91	164	PCR-RFLP
Park SS (32)	2003	Korea	Asian	58	79	PCR-RFLP
Cheng SL (14)	2004	China	Asian	184	212	PCR-RFLP
Xiao D (15)	2004	China	Asian	100	100	PCR-RFLP
Hersh CP (16)	2005	USA	Caucasian	304	441	Taqman
Park JY (29)	2005	USA	Caucasian	131	262	PCR-RFLP
Brøgger J (17)	2006	Norway	Caucasian	248	244	Taqman
Fu WP (18)	2006	China	Asian	256	266	Sequencing and PCR-RFLP
Matheson MC (31)	2006	Australia	Caucasian	72	220	ARMS
Vibhuti A (19)	2007	India	Asian	202	136	PCR-RFLP
Chappell S (23)	2008	European countries	Caucasian	1,017	912	Taqman
Zidzik J (22)	2008	Slovakia	Caucasian	217	160	PCR-RFLP
Lakhdar R (24)	2009	Tunisia	African	234	182	PCR-RFLP
Zheng Q (21)	2009	China	Asian	80	87	PCR-RFLP
Penyige A (25)	2010	Hungary	Caucasian	272	301	Taqman
Chen CZ (27)	2011	China	Asian	105	103	PCR-RFLP
Lee J (26)	2011	Denmark	Caucasian	4,127	37,964	Taqman

**Table II:** Summary ORs for the association of the EPHX1 113 mutant homozygote and heterozygote with COPD risk.

Study group	N	113CC vs. TT OR (95% CI)	P-value	P-value for heterogeneity	I^2^ (%)	113CT vs. TT OR (95% CI)	P-value	P-value for heterogeneity	I^2^ (%)
Total	25	1.33 (1.06–1.69)	**0.02**	<0.0001	73	1.12 (0.96–1.30)	0.14	<0.00001	69
Ethnicity									
Asian	12	1.07 (0.76–1.52)	0.69	0.002	62	1.07 (0.69–1.65)	0.77	<0.00001	80
Caucasian	12	1.61 (1.12–2.31)	**0.01**	<0.0001	81	1.08 (0.96–1.22)	0.20	0.10	37
Sample size									
>200	19	1.54 (1.17–2.02)	**0.002**	<0.0001	78	1.16 (0.98–1.37)	0.09	<0.00001	75
<200	6	0.80 (0.55–1.15)	0.23	0.76	0	0.92 (0.64–1.33)	0.67	0.35	11
Smoking status									
Smokers	14	1.11 (0.84–1.48)	0.46	0.0005	64	1.06 (0.82–1.38)	0.65	<0.00001	77
Non-smokers	11	1.86 (1.14–3.04)	**0.01**	<0.00001	81	1.12 (0.93–1.35)	0.23	0.06	44
Controls in HWE	15	1.07 (0.86–1.34)	0.52	0.004	56	0.98 (0.86–1.12)	0.76	0.03	45
Asian	6	0.86 (0.52–1.43)	0.57	0.04	56	0.75 (0.45–1.22)	0.25	0.01	65
Caucasian	8	1.09 (0.85–1.39)	0.51	0.03	55	1.02 (0.93–1.12)	0.71	0.28	18

The bold values indicate a significant association. N, number of the studies; EPHX1, microsomal epoxide hydrolase; COPD, chronic obstructive pulmonary disease.

**Table III:** Summary ORs for the association of the putative EPHX1 enzyme activity (extremely slow and slow enzyme activity) with COPD risk.

Study group	N	Extremely slow vs. normal OR (95% CI)	P-value	P-value for heterogeneity	I^2^ (%)	Slow vs. normal OR (95% CI)	P-value	P-value for heterogeneity	I^2^ (%)
Total	15	1.77 (1.23–2.55)	**0.002**	<0.0001	76	1.44 (1.13–1.85)	**0.004**	<0.00001	74
Ethnicity									
Asian	6	1.14 (0.84–1.54)	0.40	0.95	0	1.41 (0.90–2.19)	0.13	0.03	59
Caucasian	8	2.64 (1.30–5.38)	**0.007**	<0.0001	85	1.31 (1.01–1.71)	**0.04**	0.01	62
Sample size									
>200	13	1.90 (1.25–2.88)	**0.002**	<0.0001	79	1.44 (1.11–1.88)	**0.007**	<0.00001	77
<200	2	1.21 (0.65–2.24)	0.55	0.90	0	1.39 (0.66–2.92)	0.38	0.47	0
Smoking status									
Smokers	6	1.04 (0.77–1.41)	0.80	0.73	0	1.49 (1.01–2.21)	**0.04**	0.04	58
Non-smokers	9	2.95 (1.56–5.56)	**0.00008**	<0.00001	85	1.42 (1.04–1.93)	**0.03**	0.003	73
Controls in HWE	8	1.35 (0.93–1.96)	0.11	0.01	61	1.04 (0.96–1.12)	0.36	0.0005	73
Asian	3	1.12 (0.68–1.84)	0.66	0.91	0	0.87 (0.51–1.47)	0.60	0.13	51
Caucasian	4	1.18 (0.75–1.86)	0.47	0.09	53	1.01 (0.94–1.09)	0.72	0.14	45

The bold values indicate a significant association. N, number of the studies; EPHX1, microsomal epoxide hydrolase; COPD, chronic obstructive pulmonary disease.
